# The burden of colorectal cancer survivors in the Netherlands: costs, utilities, and associated patient characteristics

**DOI:** 10.1007/s11764-021-01096-6

**Published:** 2021-09-11

**Authors:** Frederike E. C. M. Mulder, Eline H. van Roekel, Martijn J. L. Bours, Matty P. Weijenberg, Silvia M. A. A. Evers

**Affiliations:** 1 Department of Epidemiology, GROW School for Oncology and Developmental Biology, Maastricht University, Maastricht, The Netherlands; 2 Department of Health Services Research, CAPHRI Care and Public Health Research Institute, Maastricht University, Maastricht, The Netherlands

**Keywords:** Colorectal cancer, Survivorship, Societal costs, Burden of disease, Cost of illness, Quality of life, EQ-5D

## Abstract

**Purpose:**

The aim of this study is to assess the societal burden of colorectal cancer (CRC) survivorship 2–10 years post-diagnosis in terms of (1) societal costs, and (2) quality of life/utilities, and to analyze associated patient characteristics.

**Methods:**

This is a cross-sectional, bottom-up prevalence-based burden of disease study, conducted from a societal perspective in the Netherlands. In total, 155 CRC survivors were included. Utilities were measured by the EQ-5D-5L, using the Dutch tariffs. A cost questionnaire was developed to obtain cost information. Subgroup analyses were performed, based on patient characteristics and sensitivity analyses.

**Results:**

Of all CRC survivors, 81(54%) reported no problems for mobility, 133(88%) for self-care, 98(65%) for daily activities, 59(39%) for pain/discomfort, and 112(74%) for anxiety/depression on the EQ-5D-5L. The average EQ-5D-5L utility score was 0.82 (SD = 0.2) on a scale from 0 (death) to 1 (perfect health). Significant differences in utility score were found for gender, tumor stage, number of comorbidities, and lifestyle score. The average societal costs per CRC survivor per 6 months were estimated at €971 (min = €0, max = €32,425). Significant differences in costs were found for the number of comorbidities.

**Conclusions:**

This study shows a considerable burden of CRC survivors 2–10 years after diagnosis, in comparison with survivors sooner after diagnosis and with healthy individuals in the Netherlands.

**Implications for Cancer Survivors:**

Long-term care of CRC survivors should focus on improving the societal burden by identifying modifiable factors, as summarized in the WCRF/AICR lifestyle score, including body composition, physical activity, and diet.

## Introduction

Worldwide, the number of colorectal cancer (CRC) survivors is rising, and continuing growth is expected [1–3]. In 2020, over 5.25 million individuals worldwide were estimated to live with a diagnosis of colorectal cancer made in the past five years [4]. Incidence and survival are increasing predominantly due to the aging of the population, technological developments, such as population screening and improved treatment options, and changes in lifestyle factors [5]. Most CRC survivors are elderly individuals with a high risk of recurrence and up to 80% suffer from one or more comorbidities [6, 7]. Additionally, the increasing incidence of CRC in younger adults is a newly arising trend [8]. The introduction of more successful treatments has also increased long-term side effects (e.g. fatigue, peripheral neuropathy, gastrointestinal problems, urinary incontinence, and sexual dysfunction) [6, 9]. Therefore, survivors continue to require care long after diagnosis, which puts constraints on survivors, their family, society, and economy [10, 11].

The burden of disease is often estimated on societal costs and quality of life (QoL) [12, 13]. The annual burden of CRC survivors in the USA seems higher than survivors of breast and prostate cancer [14]. Additionally, the socio-economic status of CRC survivors appears to be more variable than that of breast or prostate cancer survivors, implying that costs are substantially different [11]. Most estimates of CRC costs are based on health services costs for managing the disease and societal costs of premature cancer-related mortality [11, 15]. Furthermore, there is increasing evidence that cancer survivors incur considerable cancer-related time and out-of-pocket costs and lifelong time and travel costs [10, 16], warranting the analysis of societal costs.

Because the long-term survival of CRC patients has risen substantially in the last few decades, there is growing interest in this population’s quality of life [17]. CRC survivors show decreases in social, role, emotional, cognitive, and physical functioning [17, 18]. It is suggested that QoL and symptoms might differ considerably between short-term and long-term survivors [19–22].

Further, little is known about the influence of patient characteristics on the societal burden of CRC survivors [23]. Previous studies found associations between costs and gender, age, tumor stage, comorbidities, tumor subsite, and time since diagnosis [10, 15, 24–26]. Lifestyle factors relevant to the risk of cancer (body composition, physical activity, diet) are summarized in a lifestyle score according to the cancer prevention recommendations of the World Cancer Research Fund (WCRF)/American Institute for Cancer Research (AICR) [1]. The association between this lifestyle score and costs/utility scores in CRC survivors is unknown. A higher WCRF/AICR lifestyle score was found to be associated with better physical functioning and less fatigue [1, 27]. Finding associations between the WCRF/AICR lifestyle score and costs or QoL/utilities might introduce new and early intervention methods in clinical practice, due to its modifiable nature.

The Dutch healthcare system consists of mandatory health insurance for Dutch citizens from private insurers, voluntary complementary insurance, and tax-funded, income-dependent long-term care by the government. Since 2006, citizens are able to freely choose private insurers for the mandatory health insurance, thereby introducing market competition [28]. The content of the mandatory health insurance is determined by the Dutch government, whereas insurers and care providers collectively establish prices. Furthermore, insurers offer voluntary complementary insurance, such as additional physical therapy or dentist care. Since 2015, long-term care for patients complying to the legislative conditions as confirmed by the Care Needs Assessment Centre (CIZ) is financed by income-dependent taxes [29].

To our knowledge, there is no information on Dutch societal costs of long-term CRC survivorship. Worldwide, few studies have analyzed CRC costs from a societal perspective and methodological heterogeneity and lacking transparency are present [30, 31]. Therefore, the aim of this study is to assess the burden of CRC survivors 2–10 years post-diagnosis in terms of (1) societal costs, and (2) QoL/utilities, and to analyze associated patient characteristics, including sociodemographic, clinical and lifestyle characteristics.

## Methods

This is a cross-sectional, bottom-up, prevalence-based burden of disease study from a societal perspective in the Netherlands. The study is embedded in the cross-sectional part of the “Energy for life after ColoRectal cancer” (EnCoRe) study, which assesses lifestyle and QoL of CRC survivors. The methods of the EnCoRe study have been published and are briefly described below [23].

### Setting, participants, and procedure

This study consists of patients (> 18 years of age) who have been diagnosed with and treated for stage Ι-ΙΙΙ CRC at Maastricht University Medical Center + (MUMC +) between 2002 and 2010. Patients were identified through the Netherlands Cancer Registry and recruited by mail between May 2012 and December 2013. Exclusion criteria were: (1) stage ΙV disease, (2) passed away, (3) currently no home address in the Netherlands, (4) unable to comprehend the Dutch language, and (5) presence of comorbidities that could obstruct participation. In total, 155 individuals participated in this study. The EnCoRe study was approved by the Medical Ethics Committee of MUMC + and Maastricht University, the Netherlands, and written informed consent was obtained from all participants. Participants underwent several measurements at one point during a house visit by a trained research assistant. Measurements included a general questionnaire, which was developed based on existing questionnaires by the EnCoRe research team and field experts. Additionally, the questionnaire contained questions on medical care, with recall periods of 3/6 months, depending on the estimated frequency of attendance. If estimated attendance for an activity was high, for instance visiting the general practitioner (GP), a recall period of 3 months was chosen to increase reliability. Participants wrote down all medication and supplements used in the past 6 months. The supplement packaging was checked by the research assistant.

### Measurements

The main outcome measurements are societal costs (in 2014 Euros) and QoL/utilities. A cost questionnaire was developed by field experts, based on the steps mentioned by Thorn, and pilot tested [32]. QoL was assessed with the European Quality of Life-5 Dimensions-5 Levels (EQ-5D-5L), which includes five domains (mobility, self-care, usual activities, pain-discomfort, and anxiety/depression). Each domain consists of 5 options/levels, ranging from 1 to 5 [33]. The reliability and validity of the EQ-5D-5L in cancer patients has been shown [34–36]. The EQ-5D-5L generates a five-dimension health state, which was transformed into a single utility score based on Versteegh et al. [33, 37]. The Dutch tariff showed a single utility score ranging from − 0.446 (worse-than-dead) to 0 (death) to 1 (perfect health) [36–38].

### Cost analysis and valuation

Costs of individual survivors were calculated for the six months preceding the measurement and were summed (bottom-up approach). Costs were divided into healthcare sector costs, patient and family costs, and costs in other sectors. Healthcare sector costs and patient and family costs were valuated according to the most recently updated Dutch Manual for Cost Analysis in Health Care Research from 2015 [39]. Since this is the most recent Dutch costing manual and the data were collected between 2012 and 2013, all costs are in 2014 Euros. As recommended by this manual, the medication costs were based on www.medicijnkosten.nl and used the price per dose of the drug. If no start- and/or end-date of medication was noted, it was assumed survivors were taking the medication the full 6 months. If the frequency was missing, the lowest entered number by other survivors was assumed (0.5 unit). In case of missing data, the lowest price of the medication was assumed (e.g. lowest dose and cheapest brand). A standard price for supplements was estimated by calculating an average price per supplement from all house-brand supplements offered online by a Dutch store [40]. Medication and supplement prices were transformed from 2016 Euros into estimated 2014 Euros (decrease of 0.2% according to the Dutch Central Bureau of Statistics) [41]. Informal care prices were based on shadow prices (€14/h in 2014) [42]. Travel expenses and productivity losses were calculated according to the updated Dutch manual [39]. Travel expenses were estimated based on the mean distance from a house to a care organization, in kilometers multiplied by the standard cost price per kilometer (€0.19). The friction cost method was used for productivity losses, which multiplies the days of production lost till replacement (85 days) by the average day-wage (€34.75). Conservative estimates (lowest cost price) were used in case of uncertainty.

### Statistical methods

Survivors were excluded from the analyses if > 1 item on the EQ-5D-5L was missing. In case of one missing value for the EQ-5D-5L, the population mean was imputed. When medical care questions were missing, the lowest entered population number, excluding zero, was imputed. However, if the total population entered a zero, a zero was imputed. The statistical analyses were performed with SPSS version 25. Seven subgroup analyses for costs and utilities were performed, namely for: gender (male/female), age (< 70/ ≥ 70 years), tumor stage (Stage I/II/III), comorbidities (0/1/ ≥ 2), tumor subsite (colon/rectosigmoid/rectum), WCRF/AICR lifestyle score (low/medium/high; based on tertiles) [1], and time since diagnosis (< 5/ ≥ 5 years). Utility score differences between subgroups were tested for significance by the Mann–Whitney U test, since the data were not normally distributed. Cost differences were tested by non-parametric bootstrapping, simulating 1000 bootstraps to estimate the total cost difference. This method is recommended in literature for analyzing skewed cost data by analyzing arithmetic means and avoiding specific distributional assumptions [43, 44]. The critical p-value was set at 0.05.

Economic evaluations are accompanied by uncertainty. In order for policy makers to correctly interpret the findings it is essential that the uncertainty of point estimates is explored [45]. Three types of sensitivity analyses were performed [46]: (1) using the UK value set to derive utility scores from the EQ-5D-5L and comparing this to the utility scores derived from the Dutch value set, (2) comparing the outcomes of all cases versus all complete cases (no missing data), and (3) removing total cost outliers (≥ 3 SD) from the analyses.

## Results

Data were collected from 155 colorectal cancer survivors. Four survivors were excluded, because of > 1 missing item on the EQ-5D-5L. The majority of the resulting 151 participants were male (62.3%), with a mean age of 70 years (SD = 8.7), and mean time since diagnosis of 5.7 years (SD = 1.8). The distribution of tumor stage was: 27.8% Stage Ι, 34.4% Stage ΙΙ, and 32.5% Stage ΙΙΙ. Just over half of survivors presented with 2 or more comorbid conditions (50.3%). Of all participants, 53.0% had a history of colon cancer, 4.6% had a rectosigmoid tumor, and 42.4% had rectal cancer (Table 1).

**Table 1:** Socio-demographic and clinical characteristics of Dutch Colorectal Cancer (CRC) survivors 2–10 years post-diagnosis

	Mean (SD)/Number (%)	N
Age (years), mean (SD)	70 (8.7)	151
Gender, n (%)		151
Men	94 (62.3)	
Women	57 (37.7)	
Education level^a^, n (%)		151
Low	37 (24.5)	
Medium	52 (34.4)	
High	62 (41.1)	
Years since diagnosis, mean (SD)	5.7 (1.8)	151
Cancer stage^b^, n (%)		143
Ι	42 (27.8)	
ΙΙ	52 (34.4)	
ΙΙΙ	49 (32.5)	
Number of comorbid conditions^c^, n (%)		150
None	37 (24.5)	
1	37 (24.5)	
≥ 2	76 (50.3)	
Tumor subsite, n (%)		151
Colon	80 (53.0)	
Rectosigmoid	7 (4.6)	
Rectum	64 (42.4)	
Adherence WCRF/AICR^d^, score n (%)		148
Low	46 (31.1)	
Medium	54 (36.5)	
High	48 (32.4)	

^a^ Education level: low (none, primary education, lower vocational training), medium (lower general secondary education, intermediate vocational education), high (pre-university education, higher professional education, higher education university)

^b^ Cancer stage: Ι (T1-2 and N0 and M0), ΙΙ (T3-4 and N0 and M0), ΙΙΙ (Any T and N1-2 and M0)

^c^ Comorbidities: heart condition; stroke; high blood pressure; asthma, chronic bronchitis, COPD; diabetes; stomach ulcer; kidney disease; liver disease; anemia or other disease of the blood; thyroid gland disease; depression; osteoarthritis; back pain; rheumatic arthritis; polyps, adenomas; other comorbidities

^d^ World Cancer Research Fund (WCRF)/American Institute for Cancer Research (AICR) lifestyle score, according to tertiles

### Quality of life and utility scores

Survivors showed, on a scale from 1 to 5 on the EQ-5D-5L subscales, mean values of 1.9 for mobility (SD = 1.0), 1.2 for self-care (SD = 0.7), 1.6 for daily activities (SD = 0.9), 1.9 for pain/discomfort (SD = 0.9), and 1.3 for anxiety/depression (SD = 0.6). The average EQ-5D-5L utility score was 0.8 (SD = 0.2) (Table 2). Males had a significantly higher utility score (0.85; SD = 0.2) than females (0.77; SD = 0.2). Furthermore, stage ΙΙΙ survivors had a significantly higher utility score (0.84; SD = 0.2) than stage Ι survivors (0.78; SD = 0.2). Survivors with two or more comorbidities had significantly lower utility scores (0.75; SD = 0.2) than survivors having one (0.88; SD = 0.1) or zero (0.92; SD = 0.09) comorbidities. Survivors with a low WCRF/AICR lifestyle score had a significantly lower utility score (0.78; SD = 0.2) than those with a medium score (0.82; SD = 0.2), or a high score (0.86; SD = 0.1). No significant differences in utility score for age, tumor subsite, or time since diagnosis were found (Table 3).

**Table 2:** EQ-5D-5L and utility scores in Dutch Colorectal Cancer (CRC) survivors 2–10 years post-diagnosis (n = 151)

EQ-5D-5L	No problems, n (%)	Mean	SD	Min	Max
Mobility (1–5)	81 (54%)	1.85	1.0	1	4
Self-care (1–5)	133 (88%)	1.22	0.7	1	5
Daily activities (1–5)	98 (65%)	1.56	0.9	1	5
Pain/discomfort (1–5)	59 (39%)	1.89	0.9	1	4
Anxiety/depression (1–5)	112 (74%)	1.34	0.6	1	3
Utility score	-	0.82	0.2	− 0.1	1.0

**Table 3:** Subgroup utility scores in Dutch Colorectal Cancer (CRC) survivors 2–10 years post-diagnosis. Statistical significance tested using the Mann–Whitney U test

	Utility scores (0–1)			
Characteristics	Mean	SD	N	Sign
Gender (151)				
Men	0.85	0.16	94	p = 0.016
Women	0.78	0.21	57	
Age (151)				
< 70 years	0.83	0.15	79	p = 0.753
≥ 70 years	0.81	0.21	72	
Tumor stage (143)				
Stage I	0.78	0.17	42	I-II p = 0.112 I-III p = 0.041 II-III p = 0.690
Stage II	0.82	0.21	52	
Stage III	0.84	0.17	49	
Comorbidities (150)				
0	0.92	0.09	37	0–1 p = 0.307 0–2 p = 0.000 1–2 p = 0.000
1	0.88	0.13	37	
≥ 2	0.75	0.21	76	
Tumor subsite (151)				
Colon	0.80	0.21	80	p = 0.172
Rectosigmoid/rectum	0.85	0.14	71	
WCRF/AICR score (148)				
Low	0.78	0.18	46	L-M p = 0.046 L–H p = 0.021 M-H p = 0.861
Medium	0.82	0.22	54	
High	0.86	0.14	48	
Time since diagnosis (151)				
< 5 years	0.84	0.14	46	p = 0.644
≥ 5 years	0.81	0.20	105	

### Resource use and societal costs

**Fig. 1:**
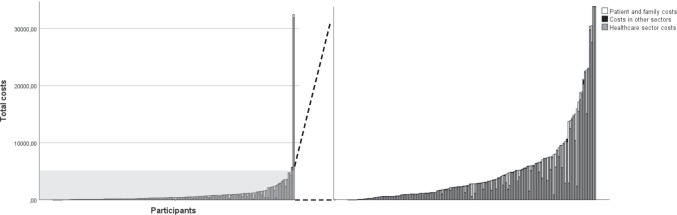
Total costs in 2014 Euros of individual participants divided by (1) healthcare sector costs (gray), (2) patient and family costs (white), and (3) costs in other sectors (black)

The resource use categories that showed the highest absolute number of users were medication (77.5%), travel costs (81.5%), and medical specialist (64.9%). The largest resource use per average patient was for paramedical care, with a mean of 2.9 (SD = 8.3). The estimated average societal costs per CRC survivor per 6 months were €971 (min = €0, max = €32,425). The highest costs per average survivor were observed for the categories nursing home (€204), medication (€193), and medical specialist (€141). Survivors with a time since diagnosis of ≥ 5 years showed higher total societal costs (€1007) compared to survivors with a time since diagnosis < 5 years (€888). The largest differences in costs between these two groups were for hospital and nursing home costs. Overall, the healthcare sector costs contained the largest mean costs per average patient (€849), followed by patient and family costs (€120), and then costs in other sectors (€2) (Table 4; Fig. 1).

**Table 4:** Total societal costs in 6 months of Dutch Colorectal Cancer (CRC) survivors 2–10 years post-diagnosis in 2014 Euros (n = 151)

	Resource use			Costs				
	Absolute number of users N (%)	Resource use per average patient		Costs per average patient in 2014 Euros			*Costs for* < *5 years survivors* *in 2014 Euros*	*Costs for* ≥ *5 years survivors* *in 2014 Euros*
		Mean	SD	Mean	SD	Median	Mean (SD)	Mean (SD)
Healthcare sector costs								
Outpatient								
General practitioner	91 (60.3%)	1.6	2.9	102.5	193.1	66.0	114.8 (301.8)	97.1 (119.5)
Medical specialist	98 (64.9%)	1.6	2.8	140.7	242.9	86.0	175.7 (201.0)	125.3 (258.6)
Paramedical^a^	38 (25.2%)	2.9	8.3	96.8	274.1	0.0	127.7 (350.0)	83.3 (234.0)
Mental health care Professionals^b^	5 (3.3%)	0.2	1.2	16.2	108.4	0.0	0.0 (0.0)	23.3 (129.6)
Alternative worker	6 (4.0%)	0.1	0.4	5.8	29.9	0.0	5.8 (27.6)	5.7 (31.0)
Other care/advice/support^c^	3 (2.0%)	0.1	0.7	3.6	27.0	0.0	6.3 (43.0)	2.4 (15.7)
Inpatient								
Psychiatric hospital	0 (0.0%)	0.0	0.0	0.0	0.0	0.0	0.0 (0.0)	0.0 (0.0)
Hospital	5 (3.3%)	0.1	0.7	72.3	442.0	0.0	209.3 (766.3)	12.2 (125.3)
Nursing home	1 (0.7%)	1.2	14.9	203.6	2501.9	0.0	0.0 (0.0)	292.8 (3000.3)
Elderly home	0 (0.0%)	0.0	0.0	0.0	0.0	0.0	0.0 (0.0)	0.0 (0.0)
Rehabilitation center^f^	3 (2.0%)	-	-	5.9	40.0	0.0	0.0 (0.0)	8.5 (47.8)
Medication^f^	117 (77.5%)	-	-	193.1	510.7	36.5	151.5 (376.4)	211.3 (560.2)
Supplements^f^	43 (28.9%)	-	-	8.6	26.4	0.0	2.9 (7.8)	11.1 (30.9)
*Total healthcare sector costs*	*-*	*-*	*-*	*849.0*	*2704.5*	*342.2*	*794.1 (1095.2)*	*873.1 (3166.8)*
Patient and family costs								
Travel expenses^g^	124 (82.1%)	-	-	9.7	24.7	3.9	9.5 (12.2)	9.8 (28.6)
Informal care	36 (23.8%)	1.5^d^	4.3	110.4	295.6	0.0	81.7 (222.0)	123.0 (322.7)
*Total patient and family costs*	*-*	*-*	*-*	*120.1*	*297.0*	*5.7*	*91.2 (223.3)*	*132.8 (324.2)*
Costs in other sectors								
Absenteeism in paid work	4 (2.6%)	0.2^e^	1.6	1.4	10.4	0.0	2.7 (15.4)	0.9 (7.2)
Absenteeism in unpaid work	1 (0.7%)	0.0^e^	0.1	0.0	0.5	0.0	0.0 (0.0)	0.1 (0.6)
*Total costs in other sectors*	*-*	*-*	*-*	*1.5*	*10.4*	*0.0*	*2.7 (15.4)*	*0.9 (7.2)*
*Total societal costs*	*-*	*-*	*-*	*970.6*	*2761.0*	*432.7*	*888.0 (1202.0)*	*1006.8 (3219.4)*

^a^ Paramedical = physiotherapist, dietician, occupational therapist, remedial therapist, another paramedic

^b^ Mental health care professionals = psychologist/psychotherapist, sexologist, social worker, psychiatrist, another social worker for emotional or psychological complaints

^c^ Other care/advice/support = pastoral care, fellow sufferer contact, ‘Herstel & Balans’ program, creative therapy

^d^ Resource use in hours/week in the past 6 months

^e^Resource use in days/week

^f^ Mean and SD cannot be calculated due to different types of resources

^g^ Mean and SD are not calculated because of standard distances from the updated Dutch Manual for Cost Analysis in Health Care Research[39]

### Subgroup costs

Survivors with two or more comorbidities presented with significantly higher costs (€1514) than survivors having one (€528) or zero (€316) comorbidities. There were no significant differences in costs for sex, age, tumor stage, tumor subsite, and WCRF/AICR lifestyle score (Table 5).

**Table 5:** Subgroup mean total societal costs per 6 months of Dutch *Colorectal Cancer (*CRC) survivors 2–10 years post-diagnosis. Cost difference analyses were performed using non-parametric bootstrapping (1000 times)

	Costs			Difference in costs			
	Mean	SD	N	Mean Δ	SD	Median	95% CI
Gender (n = 151)							
Men	€1006	3408.2	94	-€83	€380	-€35	(-€986, €514)
Women	€912	1059.3	57				
Age (n = 151)							
70 years	€771	1075.3	79	€423	€471	€381	(-€267, €1518)
≥ 70 years	€1190	3839.4	72				
Tumor stage (143)							
Stage I	€1622	4989.9	42	I-II -€773	€804	-€676	(-€2699, €362)
Stage II	€889	1075.1	52	I-III -€959	€741	-€895	(-€2674, €58)
Stage III	€608	854.7	49	II-III -€277	€193	-€284	(-€643, €110)
Comorbidities (150)							
0	€316	367.8	37	0–1 €213	€138	€200	(-€11, €527)
1	€528	807.4	37	0–2 €1207	€441	€1154	(€579, €2236)
≥ 2	€1514	3775.4	76	1–2 €979	€444	€920	(€285, €1993)
Tumor subsite (151)							
Colon	€1188	3644.4	80	-€462	€429	-€421	(-€1401, €148)
Rectosigmoid/rectum	€726	1109.3	71				
WCRF/AICR^a^ score (148)							
Low (L)	€837	914.6	46	L-M €577	€638	€497	(-€287 – €2046)
Medium (M)	€1409	4453.9	54	L–H -€216	€177	-€215	(-€550 – €108)
High (H)	€624	886.4	48	M-H -€798	€602	-€735	(-€2153 – €119)
Time since diagnosis (151)							
< 5 years	€888	1202.0	46	€109	€349	€85	(-€497 – €844)
≥ 5 years	€1007	3219.4	105				

^a^ World Cancer Research Fund (WCRF)/American Institute for Cancer Research (AICR) lifestyle score

### Sensitivity analyses

Sensitivity analyses using the UK value set by Devlin et al. (2018) yielded a mean utility score of 0.85 (SD = 0.2; min = 0.1; max = 1.0), compared to the Dutch value set, which resulted in a utility score of 0.82 (SD = 0.2; min =  − 0.1; max = 1.0) [47]. Removing incomplete cases (n = 14), resulted in utility scores of 0.83 (SD = 0.2) (Dutch value set) and 0.86 (SD = 0.2) (UK value set), and total societal costs of €987 (SD = 2893.5). After removal of outliers (n = 1), total societal costs were €761 (SD = 994.6; min = 0.0; max = 5679.1). These analyses suggest limited influence of these variations on the outcomes, thus adding to the robustness of this study.

## Discussion

The male gender, a higher tumor stage, a lower number of comorbidities, and a higher WCRF/AICR lifestyle score were associated with higher average utility scores. The average societal costs per 6 months were €971, ranging from €0 to €32,425. Significant differences in costs were observed for having ≥ 2 comorbidities compared to one or zero.

The societal costs for CRC survivors are lower compared to the average annual health expenses of the general Dutch population in 2017 (€5100), but are higher compared to the average annual health expenses of individuals with cancer in 2017 (€343) or individuals with CRC (€35) [48, 49]. Additionally, CRC survivors 2–10 years post-diagnosis showed slightly lower utility scores compared to the general Dutch population (0.87) [37], lower utility scores than patients prior to CRC surgery in the Netherlands (0.88) [50], and higher utility scores compared to CRC patients in the primary treatment phase [50].

The highly variable costs are in accordance with previous studies, where the majority of cancer survivors had little or no costs and a small number incurred very high costs [51]. The mean costs in this population were hypothesized to be lower than costs in the primary treatment phase, which several studies have demonstrated to be associated with highest costs [50, 52]. Interestingly, the mean costs of this study were only slightly lower than the costs of rehabilitation (€2106, 6–18 months from diagnosis) and remission (€2812, > 18 months from diagnosis) phase as demonstrated by Färkkilä et al. [52]. This suggests that the societal costs of long-term survivors do not substantially decrease over the years. Additionally, in accordance with Färkkilä et al., survivors ≥ 5 years post-diagnosis demonstrate higher societal costs than survivors < 5 years post-diagnosis [52]. The higher spending by long-term survivors might be explained by their comorbidity burden [53]. A number of studies have suggested an association between the number of comorbidities and costs [14, 54, 55], however, others suggest this association is limited [15, 56]. This study adds to the evidence suggesting an association between comorbidities and costs. The fact that a substantial percentage of survivors in this population presented with two or more comorbidities (50.3%) and the observation that this subgroup showed considerably higher costs and a higher standard deviation than those with zero or one comorbidities suggests that the presence of comorbidities may explain the highly variable costs in the total population.

The QoL in this population appeared to be relatively high compared to the general population [37]. It is well-described in literature that long-term CRC survivors are able to achieve similar QoL scores compared to the general population [57–60]. Plausible explanations for the high QoL in this population are posttraumatic emotional growth of survivors and positive changes due to the recovery of a possibly fatal condition. Therefore, the comparison of the QoL of cancer survivors with healthy individuals is difficult because of the potential response shift (lowered expectations and a decrease in capabilities might adjust standards) [61]. Quality of life of CRC survivors might also be impacted by improved coping mechanisms and altruism due to positive adaptation, by evaluating personal experiences and goals [62]. The significantly higher utility score in males (0.85) compared to females (0.77) is consistent with the results of Versteegh et al. [37]. Pattamatta et al. state that males oftentimes score their health better in comparison with females [50]. It is suggested that this gender difference in quality of life is explained by lower income, lower educational level, increased household responsibilities, and increased comorbidities of females compared to males [63–65]. Additionally, survivors of a stage III tumor showed a significantly higher utility score than survivors of a stage I tumor. A review of the association between tumor stage and QoL demonstrated inconclusive results [66]. The same review also showed strong evidence for comorbidities as a predictor for QoL [66], which is in line with the results of the current study. It should be noted that comparison of utility score studies is complicated by different tariffs that are used for the EQ-5D-5L. This is the first study to have explored the association between the WCRF/AICR lifestyle score and costs/utilities, suggesting that lifestyle, as a modifiable factor, might be of great value in the long-term care of CRC survivors. Additionally, it raises the question whether survivors who improve their WCRF/AICR lifestyle score might thereby improve their utility score.

Strengths of this study are the inclusion of all societal costs, including healthcare sector costs, patient and family costs, and costs in other sectors. Also, missing data is limited. Limitations of the study are, first, measurements were performed at one point in time due to the cross-sectional design. Due to this design, no causal relationship can be established, and quality of life and costs cannot be studied over time [1]. Second, collected data were mostly self-reported and were therefore prone to under- or over-reporting. However, Noben et al. reported that self-reported data can present an adequate estimate of healthcare use [67]. Third, the recall period of 3/6 months can lead to recall bias. Finally, cost calculations were performed based on the Dutch healthcare system and transferability of costs to different healthcare systems should be considered with caution.

In conclusion, this study demonstrates the considerable societal burden of CRC survivors in the Netherlands long after diagnosis. Interestingly, an association between the WCRF/AICR lifestyle score and utility score was found, implying a possible role for lifestyle factors in relation to the burden of CRC survivors. Future studies should focus on replicating these findings in a longitudinal design and study the association between lifestyle scores and cost/utilities. Additionally, future studies should further explore variables, such as particular comorbidities or medical oncologic therapy, that might explain cost/utility differences.
